# NIA Division of Aging Biology

**DOI:** 10.1093/geroni/igaf122.1559

**Published:** 2025-12-31

**Authors:** Viviana Perez Montes

**Affiliations:** National Institute on Aging, Bethesda, Maryland, United States

## Abstract

Dr. Perez Montes will discuss research foci of the NIA Division of Aging Biology. Dr. Perez Montes will also be available for small group discussion.

